# How Does a Public Health Emergency Motivate People’s Impulsive Consumption? An Empirical Study during the COVID-19 Outbreak in China

**DOI:** 10.3390/ijerph17145019

**Published:** 2020-07-13

**Authors:** Mo Li, Taiyang Zhao, Ershuai Huang, Jianan Li

**Affiliations:** 1 School of International, Jilin University of Finance and Economics, Changchun 130012, China; jasmine@jlufe.edu.cn; 2 School of Philosophy and Sociology, Jilin University, Changchun 130012, China; 3 Business School, Jilin University, Changchun 130012, China; huanges18@mails.jlu.edu.cn (E.H.); ljn18@mails.jlu.edu.cn (J.L.)

**Keywords:** public health emergency, impulsive consumption, perceived control, materialism, COVID-19

## Abstract

Impulsive consumption is a typical behavior that people often present during public health emergencies, which usually leads to negative outcomes. This study investigates how public health emergencies, such as COVID-19, affect people’s impulsive consumption behavior. Data from 1548 individuals in China during the COVID-19 outbreak was collected. The sample covered 297 prefecture-level cities in 31 provincial administrative regions. The research method included the use of a structural equation model to test multiple research hypotheses. The study finds that the severity of a pandemic positively affects people’s impulsive consumption. Specifically, the more severe the pandemic, the more likely people are to make impulsive consumption choices. The results indicate that both perceived control and materialism play mediating roles between the severity of a pandemic and impulsive consumption. As conclusions, people’s impulsive consumption during public health emergencies can be weakened either by enhancing their perceived control or by reducing their materialistic tendency. These conclusions are valuable and useful for a government’s crisis response and disaster risk management.

## 1. Introduction

The effects of the COVID-19 pandemic is global. In this global public health emergency, people are confronted with mixed information and are uncertain of what the future holds. Impulsive consumption behavior in public emergencies is widespread and universal and, consciously or unconsciously, results in irrational consumption behavior. For example, Australian residents rushed to supermarkets to stock up on necessary supplies resulting in toilet paper being sold out [1]. Furthermore, a supermarket in San Francisco was flooded with more than 500 customers within 30 minutes after it opened, and almost all the bottled water was purchased within hours [2]. This panic occurred throughout the United States, where residents scrambled to buy hand sanitizers, antiseptic wipes, surgical masks, and toilet paper. Similarly, in the United Kingdom, residents stocked up on a large amount of fresh food resulting in food waste [3]. This study focuses on the irrational consumption phenomenon, namely impulsive consumption, displayed during such a pandemic.

The impulsive consumption behavior of residents buying up certain commodities can be observed during both the SARS (Severe Acute Respiratory Syndrome) outbreak and as a result of the Fukushima nuclear power plant leakage [4]. Additionally, impulsive consumption behavior is observed with regard to non-essential goods as well. Ammo, as one of the largest online gun shopping platform in the United States, saw a 309% increase in revenue and a 222% increase in quantity of orders in February 2020 [5]. Therefore, this study considers the impulsive consumption decision of all commodities during the COVID-19 pandemic.

Impulsive consumption behavior in emergencies has multiple negative outcomes. Although people impulsively purchase various products in times of crisis, it does not limit the possible threat people face but also creates panic and results in management costs to society. As a result, products purchased tend to not be consumed, leading to waste and often a financial burden. Furthermore, impulsive consumption increases pressure on suppliers to satisfy demand during a pandemic. Additionally, individuals are influenced at a local level by their surrounding community, sometimes even accompanied by rumors and mass disturbances, which poses a potential threat to social stability.

Although impulsive consumption is a worldwide phenomenon, limited studies identify the underlying psychological causes. This study analyzes the psychological mechanism and the underlying logic of impulsive consumption behavior during the COVID-19 pandemic. This study collected primary data in February 2020 from 1548 individuals in China during the outbreak of COVID-19. This study contributes toward research on advancing disaster risk response plans and provides examples of managing public social emergencies.

## 2. Theoretical Background and Hypotheses

### 2.1. Impulsive Consumption in Social Emergencies

Impulsive consumption refers to consumers’ sudden, spontaneous, and unplanned behavior to purchase goods, which is characterized by quick decision-making and immediate ownership [6]. Usually, driven by the external environment or pursuit of timely pleasure, consumers take little thoughtful consideration when making impulsive consumption decisions [7]. It is found that external factors can induce impulsive consumption behavior. For example, research indicates that people are more likely to react with impulsive consuming behavior when stimulated by a shopping atmosphere [8]. However, previous research on impulsive consumption has mainly focused on behavior marketing situations or online shopping [9,10,11]. Thus, there are limited data available on social situations during certain emergencies. This study focuses on whether the COVID-19 pandemic, as a public health emergency, motivates people’s impulsive consumption behavior.

The outbreak of the COVID-19 pandemic may have multiple impacts on an individual, which may motivate their impulsive consumption behavior. For example, COVID-19 triggers an individual’s fear of death. Studies in consumer behavior have found that consumers may over-consume and overindulge in challenging times. The studies indicate an increase in an individual’s demand for immediate enjoyment during this time [12,13]. Moreover, during a pandemic, people are often experiencing various forms of psychological distress, such as fear and anxiety. Previous studies found that these consumers are more likely to consume impulsively to obtain immediate satisfaction to improve their mood [14]. For example, research indicates that many Americans suffered psychological trauma after the 11 September 2001 terrorist attacks. As a result, the psychological trauma correlates to increasing impulsive consumption and dependence on alcohol [15]. Based on the above logic analysis, we assume that the COVID-19 pandemic can have a positive effect on people’s impulsive consumption tendency, which would increase with the scale of the pandemic. The following hypothesis is proposed:

**Hypothesis 1** **(H1):** The severity of the pandemic is positively associated with an individual’s impulsive consumption tendency.

### 2.2. Perceived Control

Perceived control refers to individuals’ beliefs that they are able to control external factors and their environment. Perceived control differs from possessing the capacity to control one’s external environment [15]. Once an individual’s external environment is filled with uncertainty and is difficult to control, the sense of control will reduce [16]. Specifically, the COVID-19 pandemic creates an environment with a high degree of uncertainty, which leads to a decrease in the individual’s level of control. As a consequence, the more severe the pandemic, the less control one perceives. Based on the above logic analysis, the following hypothesis is proposed:

**Hypothesis 2** **(H2):** The severity of the pandemic is negatively associated with an individual’s perceived level of control.

Researchers have found that perceived control has an impact on an individual’s physical and mental health. The higher the level of perceived control an individual has, the healthier [17] and happier they will be [18]. Thus, lower levels of perceived control can cause anxiety and other negative psychological outcomes [19]. Losing control is categorized as a negative psychological experience, and as a result, individuals try and compensate for the loss. According to the compensatory control theory, individuals can increase the feeling of control by obtaining external resources [20]. Since purchasing goods is a common way for individuals to obtain external resources, purchasing may increase when an individual perceives a lack of control. Previous literature suggests that the consumption of certain products helps restore an individual’s sense of control. For example, individuals losing control are more willing to make nostalgic consumption and prefer products or logos with boundaries [21,22,23]. Liu et al. found that individuals with low perceived control are more willing to touch the products when they are making purchase decisions [24], while Peck and Childers found that touching products can increase consumers’ impulsive consumption tendency [25]. Moreover, when losing control, individuals will have a series of negative psychological reactions such as anxiety [26], which makes them more likely to react impulsively when making purchasing decisions. Based on the above logic analysis, the following hypothesis is proposed:

**Hypothesis 3** **(H3):** Perceived control is negatively associated with consumers’ impulsive consumption tendency.

### 2.3. Materialism

Materialism is a kind of value orientation that represents the extent to which individuals value possessions as an indicator of succeeding in life [27]. Materialistic individuals tend to pursue material wealth or property to achieve a sense of fulfillment and happiness [28]. Materialists mainly have the following characteristics: trying to have more than others, believing that property can bring happiness, valuing material possessions more, and desiring to obtain and keep property [29]. Although society values materialistic things [30], materialism is often perceived in a negative light. Studies have found that materialism leads to negative psychological effects on individuals. For example, research indicates that materialistic individuals are often characterized by stinginess and jealousy [31]. Moreover, these individuals tend to overspend, leaving them in debt [32].

Materialists highly value material possessions and tend to derive satisfaction from consumption [33,34]. Belk proposed that individuals with materialistic tendencies are willing to make impulsive purchases [31]. This is because they regard the acquisition of material wealth as important and as an indicator of success and the key to happiness [35,36]. Therefore, materialists are unlikely to resist the temptation to purchase when confronted with attractive products [37] and have a higher impulsive consumption tendency [38]. Some studies proved a positive correlation between the materialistic tendency and impulsive consumption of female college students [39]. Based on the above, this study proposes the following hypothesis:

**Hypothesis 4** **(H4):** Consumers’ materialism orientation is positively associated with their impulsive consumption tendency.

The outbreak of COVID-19 made people aware of how fragile life is and, as certain studies have shown, fear of death can lead to an increase in greed and materialistic tendencies [40]. Arndt et al. explained the increase in greed and the need for possessions with the use of the terror management theory [41]. Firstly, the theory indicates that individuals would enhance self-esteem to cope with the threat of death, while the findings indicate that possession and wealth could be an effective way to improve self-esteem. Secondly, individuals will cope with the threat of death with the use of psychological defense mechanisms such as their cultural worldview when faced with mortality salience [42,43]. Culture refers to the norms, beliefs, customs, laws, and habits of a specific group of people. Research indicates that the pursuit of material things has become a common cultural value of many social groups [44]. Therefore, individuals will support this cultural value to resist uneasiness in times of crisis. Based on the above, this study suggests that individuals’ materialistic tendencies will increase due to the threat of death caused by COVID-19. This study further suggests that the more serious the threat, the stronger the materialistic tendency. Based on the above, this study proposes the following hypothesis:

**Hypothesis 5** **(H5):** The severity of the pandemic is positively associated with individuals’ levels of materialism.

This study further proposes that a low level of perceived control can increase materialism. This is because losing control is a negative psychological experience, and individuals try and counter these emotions. Enriching internal or external resources can help individuals regain the sense of control [20]. Due to the supplement of internal resources needs much more time and effort, individuals are more likely to adopt the method of supplementing external resources to compensate for losing control. The main form of external resources is the material and the desire to acquisition and possession of such material resources can lead a tendency of materialism [45]. Thus, people with low perceived control may have a high materialistic tendency. Based on the above, this study proposes the following hypothesis:

**Hypothesis 6** **(H6):** Perceived control is negatively associated with consumers’ materialism.

**Figure 1:**
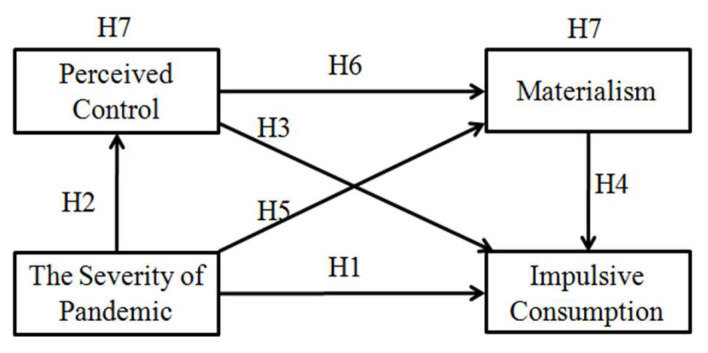
Theoretical model.

Based on the rationale above, we proposed that the severity of a pandemic could affect people’s impulsive consumption in two ways. First, the severity of a pandemic can increase the uncertainty of individuals’ external environment, reducing their perceived control [16]. Losing control makes them more likely to react impulsively when making purchasing decisions [25,26]. Second, according to terror management theory, the fear of death, which grows with the severity of a pandemic, can lead to an increase in individuals’ materialistic tendencies [40,41]. Materialism often makes people consume impulsively [38,39]. To sum up, the severity of a pandemic increases people’s impulsive consumption either by reducing their perceived control or by increasing their materialistic tendencies, that is, perceived control and materialism both mediate the effect of the severity of a pandemic on consumers’ impulsive consumption. Thus, we proposed the following theoretical model shown in Figure 1, as well as the hypothesis for the meditating effects as follows:

**Hypothesis 7** **(H7):** Perceived control and materialism can play mediating roles between the severity of the pandemic and impulsive consumption.

## 3. Methods

### 3.1. Survey Participants

Considering the social isolation policy during the COVID-19 outbreak in China, we conducted an online survey with the use of a professional platform named Credamo. We randomly sent the survey to participants on Credamo via WeChat database during February 2020. The survey consists of 1548 individuals, covering 297 prefecture-level cities in 31 provincial administrative regions in China. The demographic information of the sample is shown in Table 1. 

### 3.2. Measure

The independent variable of this study was the severity of the pandemic (SE). Because the SE varied between regions, we used data about the pandemic in the region where the participants lived as an indicator of the SE. We selected the following indicators published by the National Health Commission of the People’s Republic of China: the cumulative number of confirmed cases per province (CNP), the number of new confirmed cases per province (NNP), the cumulative number of confirmed cases per city (CNC) and the number of new confirmed cases per city (NNC). We matched the data of the survey with the above indicators according to the city and province where the participants lived during the pandemic. Specifically, using the app Credamo, we recorded the time when each respondent answered the questionnaire and positioned the city where they lived during the pandemic. Then, based on the city of each respondent, we searched the official data about the pandemic and linked it to his or her questionnaire. Since the above indicators were updated daily, we selected the indicators published on the day when the participants answered the questionnaire.

The independent variable of this study is the impulsive consumption (IC), and the meditating variables are perceived control (PC) and materialism (MA). These three variables were measured with the use of the questionnaire. As for the measurement of the IC, the previous scales mainly measure the IC behavior of consumers in malls or on online shopping platforms. As there lacks the scale measuring individuals’ IC tendency under the distinct context of the pandemic, we designed a three-item scale according to the context of this study. As for the measurement of PC, we used a three-item scale adapted from Liu et al.’s study [24]. As for the measurement of MA, an eight-item scale, based on Richins and Dawson’s study was used [27]. All of the above measurements used a 5-point Likert scale. The items of the aforementioned variables are listed in Table 2. 

We wanted to clarify which types of goods are mainly involved in the phenomenon of impulsive consumption under public health emergency. Therefore, we investigated the respondents’ impulse purchases in eight categories, namely, foods (FO), clothing (CL), daily consumables (DC), household durable products (HD), medical and health care products (MH), hedonic consumption (HC), social spending (SS), and luxury products (LP). These eight areas of consumption are considered common in Chinese daily spending patterns [46]. The respondents were asked to rate their impulse buying on each consumption item using a 5-point scale (1 = not at all, 5 = very high).

As the perceived scarcity of goods may also be an important factor influencing consumers’ impulsive consumption, we imported it as a control variable in the model. The respondents were asked to rate the extent to which they perceived the scarcity of goods on the market on a 7-point scale (1 = not at all, 7 = very scarce).

## 4. Results

### 4.1. Measurement Model, Reliability and Validity

We tested the measurement model through confirmatory factor analysis and the reliability analysis was also carried out on the variables. Considering the large sample size in this study, which might cause the chi-square expansion, we used the Bollen–Stine bootstrapping technique (5000 bootstrapping samples) for correction [47]. The results showed that the model fitting indexes of the measurement model met the minimum requirements (See Table 3). The standardized factor loading scores of each item, the average variance extracted (AVE), the composite reliability, and the Cronbach’s α for each variable are listed in Table 3. The results showed that the reliability and validity of the scales were desirable. 

The discriminant validity was examined by the correlation coefficient matrix. The intercorrelations of the variables and their AVE square roots are reported in Table 4. If the correlation coefficients between the variables are smaller than their AVE square root, it proves that the discrimination validity of the variables is desirable. As shown in Table 4, the discrimination validity of the variables in this study is good.

### 4.2. Descriptive Statistics of Impulsive Consumption Types

**Figure 2:**
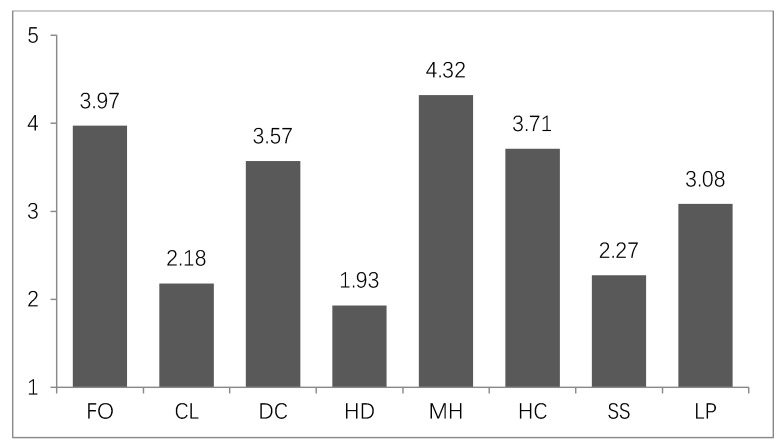
Descriptive statistics of impulsive consumption types.

As shown in Figure 2, the descriptive analysis revealed that residents’ impulses toward different types of consumption are distinctive. The item with the most impulsive consumption was medical and health care products (MH) that were the most helpful for coping with the pandemic and, thus, were scarce. Foods (FO) and daily consumables (DC) were also more impulsive consumption items, which may be due to residents wanting to reserve more resources amid the risk and uncertainty during the pandemic. Hedonic consumption (HC) and luxury products (LP) were also more impulsive consumption items. This may be because these types of item can help residents relieve negative emotions by obtaining self-satisfaction. However, residents’ consumption impulses for clothing (CL), household durable products (HD), and social spending (SS) were lower than for other items.

### 4.3. Structural Equation Model Analysis

**Figure 3:**
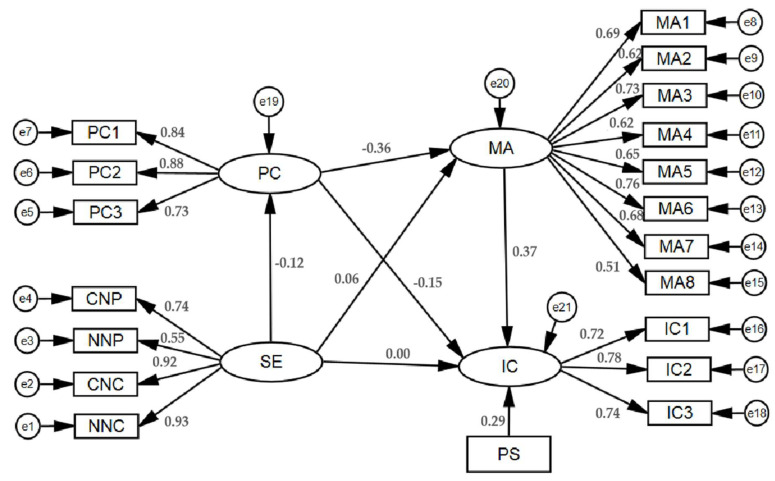
Structure equation model diagram.

Table 5 and Figure 3 present the model and estimation results of the structural equation developed in this study. The indexes of the model based on the Bollen–Stine bootstrapping technique with 5000 bootstrapping samples met the minimum requirements. The results showed that the SE has a significantly negative effect on PC (β = −0.119, *p* < 0.001). Hypothesis 2 (H2) was verified. PC has a negative effect on IC tendency (β = −0.148, *p* < 0.001). Hypothesis 3 (H3) was verified. MA has a positive effect on IC tendency significantly (β = 0.368, *p* < 0.001). Hypothesis 4 (H4) was verified. The SE has a positive effect on MA significantly (β = 0.063, *p* < 0.05). Hypothesis 5 (H5) was verified. PC has a negative effect on MA significantly (β = −0.363, *p* < 0.001). Hypothesis 6 (H6) was verified. In addition, the PS has a positive effect on IC significantly (β = 0.294, *p* < 0.001), which suggested that the perceived scarcity of goods can influence consumers’ impulsive consumption.

### 4.4. Analysis of Mediating Effect

The analysis of mediating effects was conducted using a bootstrapping procedure described by Preacher, Rucker, and Hayes [48]. This approach includes procedures that compute a 95% confidence interval (CI) for the total effect, indirect effect, and direct effect. If a CI does not include zero, it indicates that the effect is significant. The results are shown in Table 6. The total effect of the SE on the IC was significantly positive (β = 0.060, 95% CI: 0.005 to 0.118), which means the SE has a positive effect on the IC. Hypothesis 1 (H1) was verified. The indirect effect of PC and MA between the SE and the IC was significantly positive (β = 0.057, 95% CI: 0.032 to 0.083), while the direct effect was not significant (β = 0.003, 95% CI: −0.049 to 0.056). This suggests that PC and MA play fully mediating roles between the SE and the IC. Hypothesis 7 (H7) was verified.

## 5. Discussion

### 5.1. Theoretical Contributions

Most literature about the COVID-19 pandemic mainly focuses on exploring its impact on residents’ psychological state and health behaviors, such as anxiety, depression, stress, physical activity behavior, or preventive health behaviors [49,50,51,52]. Only a few studies have focused on how the COVID-19 pandemic has affected consumer behavior [53]. This study enriches and expands the research on consumption behavior during an emergency by exploring the relationship between the severity of a pandemic, perceived control, materialism, and impulsive consumption. Previous research on impulsive consumption in consumer behavior was mostly carried out in the context of shopping malls and online shopping [9,10,11]. This study highlights the idea that a public health emergency can also be an important factor that motivates consumers’ impulsive consumption. 

The findings of this study explain how public health emergencies affect individuals’ impulsive consumption. The underlying mechanisms of public health emergencies’ effect on impulsive consumption intentions were also revealed from the perspective of perceived control and materialism. Specifically, public health emergencies can reduce peoples’ perceived control, increase their materialistic tendency, and increase their impulsive consumption tendencies. The previous literature has mainly linked low perceived control to compensatory behavior that helps consumers restore their sense of control, for example, acquisition of utilitarian products and increasing their charitable contributions [54,55]. This study found impulsive consumption could be another common way for consumers to deal with feeling a low sense of control. The previous literature has mainly viewed consumers’ materialism as a function of their self-concept and cultural value [27,56]. However, the current study found that certain external social contexts such as public health emergencies, could also motivate tendencies toward materialism. Thus, the conclusions of this study can further enrich the literature of perceived control and materialism.

### 5.2. Practical Implications

The conclusions of this study are also valuable and useful for a government’s crisis response and disaster risk management. 

We found that the types of impulsive consumption during the pandemic are not limited to those related to pandemic prevention. Under the impulse of consumption motivated by the pandemic, residents often ignore the actual function of commodities and consume pervasively, which may bring them an economic burden as well as waste resources. Therefore, the government should inform the public about which products will help them deal with the pandemic and which are not necessary. We also found that perceived scarcity of goods could influence consumers’ impulsive consumption. The shortage of products during the pandemic was largely caused by impulsive and excessive purchasing by residents. Thus, the government should guide the public to restrain impulsive consumption while enhancing market supply. 

According to our study, when people perceive they are losing control during a public health emergency, they tend to become materialistic and consume impulsively. The researchers recommend that governments first improve peoples’ perception of control. The government can implement measures to regulate and control emergencies, which creates the perspective that the emergencies are being controlled and addressed accordingly. Second, the government should also restore residents’ sense of control through a variety of ways, such as by enhancing mutual assistance between communities, publicizing effective manners to deal with the pandemic situation and alleviating their inner anxiety. Moreover, media needs to provide timely and reliable information to the public. This will increase the public’s sense of control by meeting the public’s needs. 

The government should consider materialistic tendencies, which usually lead to individuals’ selfishness and indifference. The pandemic is a global health challenge that requires cooperation among citizens. Therefore, the government needs to encourage social cohesion to overcome difficulties in such times. It has been proven that helping others facilitates restoring a sense of control and reducing the tendency of materialism [55]. Therefore, the government can call on and organize residents to participate in voluntary activities or donate materials to those in need. This will not only help residents feel self-worth in helping others, but also help to deal with the pandemic objectively.

The conclusions of this study can also provide implications for businesses. First of all, consumers have strong psychological needs for items such as medical and health care products, foods, daily consumables, hedonic consumption and luxury products during the pandemic. Therefore, businesses that provide those goods and services should fully guarantee the market supply during the period. In addition, since consumers’ sense of control decrease during the pandemic, businesses can promote their products by claiming that their products can help consumers to restore or enhance their sense of control. For example, they may claim that their products can reduce the risk of infection or help people to ease anxiety.

### 5.3. Limitations and Future Research Directions

There are also some limitations to this study. First, this study only explores the mediating roles between the severity of the pandemic and impulsive consumption intention, without considering the moderators. Future research can continue to explore the boundary conditions for the current model. Second, this study examines impulsive consumption behaviors in the context of COVID-19. However, the nature and severity may differ in different kinds of emergencies. Therefore, future research can focus on various contexts to draw more general conclusions.

## 6. Conclusions

This study explores the effect of a pandemic’s severity on impulsive consumption and the corresponding underlying psychological mechanism. It has been found that the severity of the pandemic will positively affect people’s impulsive consumption; specifically, the more serious the pandemic, the more likely people are to impulsively consume. In this process, this study also confirmed that both perceived control and materialism play mediating roles between the severity of the pandemic and impulsive consumption. 

## Figures and Tables

**Table 1:** Demographic information of the sample (N = 1548).

Items	Options	Sample	Percentage	Items	Options	Sample	Percentage
Gender	Male	863	55.7%	Income per month	<3000 RMB	546	35.3%
	Female	685	44.3%		3000–6000 RMB	777	50.2%
Education level	High school or below	362	23.4%		3000–10,000 RMB	159	10.3%
	Bachelor degree	1046	67.6%		>10,000 RMB	66	4.3%
	Master degree or above	140	9%	Expense per month	<1000 RMB	344	22.2%
Age	<25	731	47.2%		1000–3000 RMB	551	35.6%
	25–40	716	46.3%		3000–5000 RMB	465	29.5%
	>40	101	6.5%		>5000 RMB	197	12.7%

**Table 2:** Items of the variables.

Variable	Item
Severity of the Pandemic (SE)	CNP: Cumulative Number of confirmed cases per Province.
	NNP: Number of New confirmed cases per Province.
	CNC: Cumulative Number of confirmed cases per City.
	NNC: Number of New confirmed cases per City.
Perceived Control (PC)	PC1: At the moment, I feel helpless. (R)
	PC2: At the moment, I feel powerless. (R)
	PC3: At the moment, I feel like I don’t have a sense of control. (R)
Materialism (MA)	MA1: I admire people who own expensive homes, cars, and clothes.
	MA2: The things I own say a lot about how well I’m doing in life.
	MA3: I like to own things that impress people.
	MA4: Buying things gives me a lot of pleasure
	MA5: I like a lot of luxury in my life.
	MA6: I’d be happier if I could afford to buy more things.
	MA7: My life would be better if I owned certain things I don’t have.
	MA8: It sometimes bothers me quite a bit that I can’t afford to buy all the things I’d like.
Impulsive Consumption (IC)	IC1: Recently, I always have the impulse to have a product immediately when I see it.
	IC2: Recently, I always have the impulse to buy some unplanned products that I didn’t intend to buy.
	IC3: Recently, I have an impulse to consumption.

Note: (R) means this item was reverse coding.

**Table 3:** Reliability and validity of the variables (N = 1548).

Variable Name	Item	Standardized Factor Loading	C.R.	AVE	Cronbach’s α
SE	CNP	0.966	0.906	0.710	0.912
	NNP	0.943			
	CNC	0.715			
	NNC	0.711			
PC	PC 1	0.842	0.857	0.668	0.852
	PC 2	0.875			
	PC 3	0.727			
MA	MA1	0.694	0.860	0.438	0.859
	MA2	0.625			
	MA3	0.734			
	MA4	0.619			
	MA5	0.647			
	MA6	0.758			
	MA7	0.674			
	MA8	0.510			
IC	IC 1	0.758	0.805	0.579	0.804
	IC 2	0.793			
	IC 3	0.731			
Model Fit: χ^2^ /df = 1.122, RMSEA = 0.009, CFI = 0.999, IFI = 0.999, TLI = 0.999					

C.R. = Composite Reliability, AVE = Average Variance Extracted, RMSEA = Root-mean-square Error of Approximation, CFI = Comparative Fit Index, IFI = Incremental Fit Index, TLI = Tucker-Lewis Index.

**Table 4:** Discriminant validity.

	SE	PC	MA	IC
SE	**0.843**			
PC	−0.142 ***	**0.817**		
MA	0.138 ***	−0.338 ***	**0.662**	
IC	0.110 ***	−0.309 ***	0.437 ***	**0.761**

Note: *** *p* < 0.001. The off-diagonal numbers are the correlations. AVE square roots are bolded on the diagonal.

**Table 5:** Path analysis results (N = 1548).

Path			Unstandardized Estimate	Standardized Estimate	S.E.	T-Value	*p*
SE	--->	PC	−0.087	−0.119	0.021	−4.126	0.000
PC	--->	MA	−0.357	−0.363	0.030	−11.766	0.000
SE	--->	MA	0.045	0.063	0.019	2.332	0.020
MA	--->	IC	0.337	0.368	0.030	11.136	0.000
SE	--->	IC	0.002	0.003	0.017	0.117	0.907
PC	--->	IC	−0.133	−0.148	0.027	−4.866	0.000
PS	--->	IC	0.201	0.294	0.018	11.082	0.000
Model Fit: χ2 /df = 1.129, RMSEA = 0.009, CFI = 0.999, IFI = 0.999, TLI = 0.998							

RMSEA = Root-mean-square Error of Approximation, CFI = Comparative Fit Index, IFI = Incremental Fit Index, TLI = Tucker-Lewis Index.

**Table 6:** The results of mediating effect. (N = 1548).

Path	Effect	Unstandardized Estimate	Standardized Estimate	S.E.	95% Confidence Intervals
SE ---> (PC and MA) ---> IC	Total effect	0.039	0.060	0.029	[0.005, 0.118]
	Indirect effect	0.037	0.057	0.013	[0.032, 0.083]
	Direct effect	0.002	0.003	0.027	[−0.049, 0.056]

## References

[1] Rumor Buster: Is it COVID-19 that Empties Supermarket Shelves of Toilet Paper?. http://www.xinhuanet.com/english/2020-03/12/c_138870183.htm.

[2] The Coronavirus Outbreak Swept over the World, Many Countries Entered a State of Emergency, Supermarkets were Robbed by People. https://xw.qq.com/cmsid/20200304A00CQG00.

[3] Britain Becomes a “City of Waste” by Hoarding 8.8 Billions of Food in Two Weeks, Where Food Piled Up in Garbage Cans Like “Hills”. https://3g.163.com/dy/article_cambrian/F8T2MB750541A1O8.html?from=history-back-list.

[4] Qi H.F. (2015). The Research into Chinese Consumers’ Impulsive Purchasing Behavior in the Situation of Emergent Accidents. J. East China Univ. Sci. Technol. (Soc. Sci. Edi.).

[5] Guns are Being Snapped Up by Americans after the Coronavirus has Confirmed Cases of 27,000. https://baijiahao.baidu.com/s?id=1661867164837104627&wfr=spider&for=pc.

[6] Hoch S.J., Loewenstein G.F. (1991). Time-inconsistent preferences and consumer self-control. J. Consum. Res..

[7] Sengupta J., Zhou R.R. (2007). Understanding impulsive eaters’ choice behaviors: The motivational influences of regulatory focus. J. Mark. Res..

[8] Morrin M., Chebat J.C. (2005). Person-place congruence: The interactive effects of shopper style and atmospherics on consumer expenditures. J. Serv. Res..

[9] Cobb C.J., Hoyer W.B. (1986). Planned versus impulse purchase behavior. J. Retail..

[10] Floh A., Madlberger M. (2013). The role of atmospheric cues in online impulse-buying behavior. Electron. Commer. Res. Appl..

[11] Chen J.V., Su B.C., Widjaja A.E. (2016). Facebook C2C social commerce: A study of online impulse buying. Decis. Support Syst..

[12] Chen S., Wei H., Meng L., Ran Y. (2019). Believing in Karma: The effect of mortality salience on excessive consumption. Front. Psychol..

[13] Friese M., Hofmann W. (2008). What would you have as a last supper? Thoughts about death influence evaluation and consumption of food products. J. Exp. Soc. Psychol..

[14] Sarah M.E., Arthur K. (2003). Negative affect the dark side of retailing. J. Bus. Res..

[15] Perrine M.B., Schroder K.E., Forester R., McGonagle-Moulton P., Huessy F. (2004). The impact of the 11 September 2001, terrorist attacks on alcohol consumption and distress: Reactions to a national trauma 300 miles from Ground Zero. J. Stud. Alcohol..

[16] Burger J. (1989). Negative reactions to increases in perceived personal control. J. Personal. Soc. Psychol..

[17] Lachman M.E., Weaver S.L. (1998). The sense of control as a moderator of social class differences in health and well-being. J. Personal. Soc. Psychol..

[18] Ruthig J.C., Chipperfield J.G., Perry R.P., Newall N.E., Swift A. (2007). Comparative risk and perceived control: Implications for psychological and physical well-being among older adults. J. Soc. Psychol..

[19] Whalen P.J. (1998). Fear, vigilance, and ambiguity: Initial neuroimaging studies of the human amygdala. Curr. Dir. Psychol. Sci..

[20] Kay A.C., Whitson J.A., Gaucher D., Galinsky A.D. (2009). Compensatory control achieving order through the mind, our institutions, and the heavens. Curr. Dir. Psychol. Sci..

[21] Chen Y., Feeley T.H. (2012). Enacted support and well-being: A test of the mediating role of perceived control. Commun. Stud..

[22] Frazier P., Keenan N., Anders S., Perera S., Shallcross S., Hintz S. (2011). Perceived past, present, and future control and adjustment to stressful life events. J. Personal. Soc. Psychol..

[23] Cutright K.M. (2012). The beauty of boundaries: When and why we seek structure in consumption. J. Consum. Res..

[24] Liu W.M., Wang J.Y., Shao J.P. (2016). An elaboration on the underlying mechanisms through which consumers’ desire for product touch forms: A cognitive experiential perspective. Acta Psychol. Sin..

[25] Peck J., Childers T.L. (2006). If I touch it I have to have it: Individual and environmental influences on impulse purchasing. J. Bus. Res..

[26] Raghunathan R., Pham M.T. (1999). All negative moods are not equal: Motivational influences of anxiety and sadness on decision making. Organ. Behav. Human Decis. Process..

[27] Richins M.L., Dawson S. (1992). A consumer values orientation for materialism and its measurement: Scale development and validation. J. Consum. Res..

[28] Chan K., Prendergast G. (2007). Materialism and social comparison among adolescents. Soc. Behav. Personal. Int. J..

[29] Ger G., Belk R.W. (1996). Cross cultural differences in materialism. J. Econ. Psychol..

[30] Mukerji C. (1983). From Graven Images: Patterns of Modern Materialism.

[31] Belk R.W. (1985). Materialism: Trait aspects of living in the material world. J. Consum. Res..

[32] Richins M.L. (2011). Materialism, transformation expectations, and spending: Implications for credit use. J. Public Policy Mark..

[33] Fournier S., Richins M.L. (1991). Some theoretical and popular notions concerning materialism. J. Soc. Behav. Personal..

[34] Kasser T., Ahuvia A. (2002). Materialistic values and well-being in business students. Eur. J. Soc. Psychol..

[35] Richins M.L. (1994). Valuing things: The public and private meanings of possessions. J. Consum. Res..

[36] Richins M.L. (2004). The material values scale: Measurement properties and development of a short form. J. Consum. Res..

[37] Badgaiyan A.J., Verma A. (2014). Intrinsic factors affecting impulsive buying behavior—Evidence from India. J. Retail. Consum. Serv..

[38] Xiao S.H., Nicholson M. (2013). A multidisciplinary cognitive behavioral framework of impulse buying: A systematic review of the literature. Int. J. Manag. Rev..

[39] Moran B., Kwak L.E. (2015). Effect of stress, materialism and external stimuli on online impulse buying. J. Res. Consum..

[40] Kasser T., Sheldon K.M. (2000). Of wealth and death: Materialism, mortality salience, and consumption behavior. Psychol. Sci..

[41] Arndt J., Solomon S., Kasser T., Sheldon K.M. (2004). The Urge to Splurge: A Terror Management Account of Materialism and Consumer Behavior. J. Consum. Psychol..

[42] Greenberg J., Solomon S., Pyszczynski T. (1997). Terror Management Theory of Self-Esteem and Cultural Worldviews: Empirical Assessments and Conceptual Refinements. Adv. Exp. Soc. Psychol..

[43] Pyszczynski T., Greenberg J., Solomon S. (1997). Why do we need what we need? A terror management perspective on roots of human social motivation. Psychol. Inq..

[44] Bauman Z. (1995). Life in Fragments: Essays in Postmodern Morality.

[45] Tatzel M. (2002). “Money worlds” and well-being: An integration of money dispositions, materialism and price-related behavior. J. Econ. Psychol..

[46] Jin X., Yang X., Wang T. (2014). Theoretical framework and empirical Study of Formation Mechanism of Collective Purchase Intentions of Migrant Workers: Effects of Self Congruence, Reference Group and Perceived Risk. Foreign Econ. Manag..

[47] Bollen K.A., Stine R.A. (1992). Bootstrapping goodness-of-fit measures in structural equation models. Sociol. Methods Res..

[48] Preacher K.J., Derek D.R., Andrew F.H. (2007). Addressing moderated mediation hypotheses: Theory, methods, and prescriptions. Multivar. Behav. Res..

[49] Nguyen H.T., Do B.N., Pham K.M., Kim G.B., Dam H.T., Nguyen T.T., Nguyen T.T., Nguyen Y.H., Sørensen K., Pleasant A. (2020). Fear of COVID-19 Scale-Associations of Its Scores with Health Literacy and Health-Related Behaviors among Medical Students. Int. J. Environ. Res. Public Health.

[50] Kim Y., Cho J. (2020). Correlation between Preventive Health Behaviors and Psycho-Social Health Based on the Leisure Activities of South Koreans in the COVID-19 Crisis. Int. J. Environ. Res. Public Health.

[51] Stanton R., To Q., Khalesi S., Williams S.L., Alley S.J., Thwaite T.L., Fenning A.S., Vandelanotte C. (2020). Depression, Anxiety and Stress during COVID-19: Associations with Changes in Physical Activity, Sleep, Tobacco and Alcohol Use in Australian Adults. Int. J. Environ. Res. Public Health.

[52] Lesser I.A., Nienhuis C.P. (2020). The Impact of COVID-19 on Physical Activity Behavior and Well-Being of Canadians. Int. J. Environ. Res. Public Health.

[53] Song W., Jin X., Gao J., Zhao T. (2020). Will Buying Follow Others Ease Their Threat of Death? An Analysis of Consumer Data during the Period of COVID-19 in China. Int. J. Environ. Res. Public Health.

[54] Chen C.Y., Lee L., Yap A.J. (2017). Control Deprivation Motivates Acquisition of Utilitarian Products. J. Consum. Res..

[55] Xu Q., Kwan C., Zhou X. (2020). Helping Yourself before Helping Others: How Sense of Control Promotes Charitable Behaviors. J. Consum. Psychol..

[56] Gil L.D., Leckie C.T., Johnson L. (2016). The impact of self on materialism among teenagers. J. Consum. Behav..

